# Distribution of age at natural menopause, age at menarche, menstrual cycle length, height and BMI in *BRCA1* and *BRCA2* pathogenic variant carriers and non-carriers: results from EMBRACE

**DOI:** 10.1186/s13058-025-02030-9

**Published:** 2025-05-21

**Authors:** Nasim Mavaddat, Debra Frost, Emily Zhao, Daniel R. Barnes, Munaza Ahmed, Julian Barwell, Angela F. Brady, Paul Brennan, Hector Conti, Jackie Cook, Harriet Copeland, Rosemarie Davidson, Alan Donaldson, Emma Douglas, David Gallagher, Rachel Hart, Louise Izatt, Zoe Kemp, Fiona Lalloo, Zosia Miedzybrodzka, Patrick J. Morrison, Jennie E. Murray, Alex Murray, Hannah Musgrave, Claire Searle, Lucy Side, Katie Snape, Vishakha Tripathi, Lisa Walker, Stephanie Archer, D. Gareth Evans, Marc Tischkowitz, Antonis C. Antoniou, Douglas F. Easton

**Affiliations:** 1 Department of Public Health and Primary Care, Centre for Cancer Genetic Epidemiology, University of Cambridge, Cambridge, UK; 2 North East Thames Regional Clinical Genetics Service, Great Ormond Street Hospital, London, UK; 3 Leicestershire, Northamptonshire and Rutland Clinical Genetics Service, University Hospitals of Leicester NHS Trust, Leicester, UK; 4 North West Thames Regional Genetics Service, London North West University Healthcare NHS Trust, London, UK; 5 Northern Genetics Service, Newcastle Upon Tyne NHS Foundation Trust, Newcastle Upon Tyne, UK; 6 All Wales Medical Genomics Services, Wrexham Maelor Hospital, Wrexham, UK; 7 Sheffield Clinical Genetics Service, Sheffield Children’s Hospital, Sheffield, UK; 8 Department Clinical Genetics, Royal Devon University Healthcare NHS Foundation Trust, Exeter, Devon UK; 9 Department of Clinical Genetics, South Glasgow University Hospitals, Glasgow, UK; 10 Clinical Genetics Department, St Michael’s Hospital, Bristol, UK; 11 West Midlands Regional Clinical Genetics Service, Birmingham Women’s Hospital, Birmingham, UK; 12 Trinity St Jame’s Cancer Institute, Cancer Genetics Service, Dublin, Ireland; 13 Liverpool Women’s Hospital Cheshire and Merseyside Genetics, Liverpool Women’s NHS Foundation Trust, Liverpool, UK; 14 Clinical Genetics, Guy’s and St. Thomas’ NHS Foundation Trust, London, UK; 15 Royal Marsden Hospital, NHS Trust, London, England UK; 16 Clinical Genetics Service, Manchester Centre for Genomic Medicine, Manchester University Hospitals Foundation Trust, Manchester, UK; 17 NHS Grampian, North of Scotland Regional Genetics Service, Aberdeen, Scotland, UK; 18 Belfast Health and Social Care Trust, Clinical Genetics Service, Belfast, Northern Ireland UK; 19 South East Scotland Clinical Genetics Service, Western General Hospital, Edinburgh, UK; 20 All Wales Medical Genomics Service, Wales Genomic Health Centre, Cardiff, UK; 21 Leeds Genomic Medicine Service, Leeds Teaching Hospitals NHS Trust, Leeds, UK; 22 Department of Clinical Genetics, Nottingham University Hospitals NHS Trust, Nottingham, UK; 23 University Hospital Southampton NHS Trust and Princess Anne Hospital, Southampton, UK; 24 Medical Genetics Unit, St George’s, University of London, London, UK; 25 Oxford Centre for Genomic Medicine, Nuffield Orthopaedic Centre, Oxford University Hospitals NHS Foundation Trust, Oxford, UK; 26 Department of Psychology, University of Cambridge, Cambridge, UK; 27 Genomic Medicine, Manchester Academic Health Sciences Centre, Division of Evolution, Infection and Genomic Science, University of Manchester, Manchester University Hospitals NHS Foundation Trust, Manchester, UK; 28 Department of Genomic Medicine, Cambridge Biomedical Research Centre, National Institute for Health Research, University of Cambridge, Cambridge, UK; 29 Department of Oncology, Centre for Cancer Genetic Epidemiology, University of Cambridge, Cambridge, UK

**Keywords:** BRCA1, BRCA2, Menopause, Menarche, Height, Body mass index, Cancer

## Abstract

**Background:**

Carriers of germline pathogenic variants (PVs) in the *BRCA1* and *BRCA2* genes are at higher risk of developing breast and ovarian cancer than the general population. It is unclear if these PVs influence other breast or ovarian cancer risk factors, including age at menopause (ANM), age at menarche (AAM), menstrual cycle length, BMI or height. There is a biological rationale for associations between *BRCA1* and *BRCA2* PVs and reproductive traits, for example involving DNA damage and repair mechanisms. The evidence for or against such associations is limited.

**Methods:**

We used data on 3,046 *BRCA1* and 3,264 *BRCA2* PV carriers, and 2,857 non-carrier female relatives of PV carriers from the Epidemiological Study of Familial Breast Cancer (EMBRACE). Associations between ANM and PV carrier status was evaluated using linear regression models allowing for censoring. AAM, menstrual cycle length, BMI, and height in carriers and non-carriers were compared using linear and multinomial logistic regression. Analyses were adjusted for potential confounders, and weighted analyses carried out to account for non-random sampling with respect to cancer status.

**Results:**

No statistically significant difference in ANM between carriers and non-carriers was observed in analyses accounting for censoring. Linear regression effect sizes for ANM were -0.002 (95%CI: -0.401, 0.397) and -0.172 (95%CI: -0.531, 0.188), for *BRCA1* and *BRCA2* PV carriers respectively, compared with non-carrier women. The distributions of AAM, menstrual cycle length and BMI were similar between PV carriers and non-carriers, but *BRCA1* PV carriers were slightly taller on average than non-carriers (0.5 cm difference, *p* = 0.003).

**Conclusion:**

Information on the distribution of cancer risk factors in PV carriers is needed for incorporating these factors into multifactorial cancer risk prediction algorithms. Contrary to previous reports, we found no evidence that *BRCA1* or *BRCA2* PV are associated with hormonal or anthropometric factors, except for a weak association with height. We highlight methodological considerations and data limitations inherent in studies aiming to address this question.

**Supplementary Information:**

The online version contains supplementary material available at 10.1186/s13058-025-02030-9.

## Introduction

Germline pathogenic variants in *BRCA1* and *BRCA2* confer high risks of breast and ovarian cancer [1]. Reproductive factors, including age at natural menopause (ANM) and age at menarche (AAM), and anthropomorphic traits including height and body mass index (BMI) are established breast and/or ovarian cancer risk factors in the general population [2]. There is evidence from observational studies and Mendelian randomisation analyses that some breast cancer risk factors in the general population are also associated with cancer risk in PV carriers [3–10]. Risk prediction algorithms, notably BOADICEA, incorporate the effects of both PVs and other risk factors to predict cancer risk [11, 12]. These algorithms depend on assumptions about the distribution of these traits in PV carriers, and well as the associated effect sizes. It is necessary therefore to evaluate empirically the underlying distribution of the relevant cancer risk factors for *BRCA1* and *BRCA2* PV carriers. Further, management of PV carriers may include recommendation on risk reducing bilateral salpingo-oophorectomy (RRSO) and the likely timing of menopause may be an important consideration for women contemplating surgery.

Age at natural menopause is normally distributed in the general population with an average age of ~ 50 years in European ancestry women. Menopause occurs between ages 40–60 years in 99% of women and before age 40 years in ~ 1% of women; women with age at menopause less than 40 years may be diagnosed with premature ovarian insufficiency, a largely monogenic trait. Certain environmental factors are associated with earlier ANM, including lower BMI, alcohol, smoking and low birth weight. Maternal obesogenic diet during pregnancy also decreases the ovarian reserve in offspring [13, 14]. ANM has a strong genetic basis, mediated by multiple genetic loci, many of which have been identified through genome-wide association analyses [14]. ANM associated SNPs are enriched for variants near genes involved in DNA damage response (DDR). As summarized in Ruth et al. [14], DDR is the primary biological pathway that regulates reproductive senescence. Declining or inefficient activity in DNA repair mechanisms leads to accelerated ovarian aging by accumulation of DNA damage, and the BRCA genes may play a role in DSB repair in ovarian aging in humans [14]. ANM associated variants include common coding variants in *BRCA1*: the alleles associated with earlier ANM are also associated with reduced *BRCA1* expression in blood [15]. These common variants have not, however, been associated with cancer risk [16]. *BRCA1* expression decreases in human ovaries with age [17], while reduced *brca1* expression in mouse models leads to reduced ovarian reserve [17]. BRCA1 directly inhibits a functional interaction with oestrogen receptor α and thus *BRCA1* variants could also affect ANM through altered oestrogen signalling [18]. The mechanistic rationale for investigating association between and ANM and *BRCA* PV status is thus also strong.

In exome-wide analysis in UK Biobank data, rare loss-of-function (LOF) variants in *BRCA1* and *BRCA2* were associated with earlier (2.63 and 1.53 years respectively) ANM compared with non-carriers, while LOF variants in *CHEK2* were associated later ANM (3.49 years difference) [14]. Rare coding variants in other DNA damage repair genes have also been associated with ANM [19]. Earlier epidemiological studies have suggested that natural menopause occurs at a younger age in *BRCA1* and *BRCA2* PV carriers compared with women from the general population [20–22], and that *BRCA1* PV carriers may have reduced ovarian reserve [23] and consequently a shortened reproductive lifespan. Other studies, however, have reported no statistically significant differences between ANM in *BRCA1/2* carriers and the general population [24]. These analyses are, however, complicated by incompleteness of data on preventative surgeries, in particular RRSO, and potential reverse causation as a diagnosis of cancer and associated treatments may also be associated with onset of menopause. Age at menarche, weight and height are also highly heritable polygenic traits, with both rare variant and polygenic influences [25–29]. A study in 1989, Jernström et al. [30], noted that *BRCA1* PV carrier patients are small for gestational age compared with their unaffected relatives.

A number of studies have investigated association between reproductive and anthropomorphic traits and cancer risk in *BRCA1* and *BRCA2* PV carriers [10, 31–33], but apart from studies of ANM and a small study of AAM comprising only 31 *BRCA1* and 11 *BRCA2* PV carriers [34], to our knowledge there are no epidemiological studies evaluating the distribution of these traits in comparable carrier and non-carrier populations. Here we used data from the Epidemiological Study of Familial Breast Cancer (EMBRACE), a large national study of PV carriers and non-carrier relatives, to evaluate differences in reproductive and anthropometric trait distributions among *BRCA1* and *BRCA2* PV carriers and non-carriers. Information on the distribution of these traits can ultimately be used to adapt risk prediction algorithms for PV carriers and may further inform our understanding of reproductive biology of female carriers of PVs in these susceptibility genes.

## Methods

### Study design and population

Participants were enrolled through an on-going nationwide study of individuals undergoing genetic testing in regional genomics centres in the United Kingdom and Ireland (EMBRACE) (https://ccge.medschl.cam.ac.uk/embrace/). EMBRACE recruits individuals who are carriers of pathogenic or likely pathogenic variants (PVs) in breast and/or ovarian cancer susceptibility genes, and their relatives. The analysis reported here included only women of self-reported White ethnicity. Women were eligible if they were at least 18 years of age at recruitment and had tested positive for a *BRCA1* or *BRCA2* PV or were non-carrier family members of PV carriers. PVs were defined according to ENIGMA/ClinGen guidelines (https://clinicalgenome.org/affiliation/50087/). The distribution of PV sub-classes (protein-truncating, missense, in-frame deletions) are shown in STable 1.

### Data collection

All study participants were invited to complete a baseline questionnaire requesting detailed information on known or suspected risk factors for breast and ovarian cancer, including family history of cancer, height, weight at age 18, current weight, reproductive history and surgical interventions including risk-reducing mastectomy (RRM) or RRSO. The questionnaires also requested information on age at last menstruation, whether the woman had had any period in the past year, the number of years/months since last menstruation, and reason(s) for periods stopping. PV carriers also completed follow-up questionnaires: however, since these were not completed by non-carriers and the primary interest was the comparison of carriers and non-carriers, only information from the baseline questionnaire was used here.

Women were considered premenopausal if they indicated at baseline questionnaire that they had had a period in the past year, or that their periods had not stopped completely, or if the ‘reason for periods stopping’ was medication or oral contraceptive use (unless 40 years or older), pregnancy or breast-feeding, unless censored earlier due to cancer diagnosis, chemotherapy or radiotherapy, RRSO or hysterectomy. For *N* = 17 women there was no information on periods stopping or reason for menopause or age at which periods stopped, these women were considered premenopausal until age at interview. STable 2 outlines numbers of women with missing information for each variable. Age at menopause for those who indicated no period in the past year or periods had stopped completely was determined by adding 1 year to ‘age at last menstruation’. Women were considered as having experienced natural menopause if the reason for periods stopping was recorded as ‘natural menopause’ (and not for any other reason such as chemotherapy, childbirth, pregnancy, breast feeding, hysterectomy, or ‘other’ (unspecified) reason), and age at menopause preceded RRSO, any cancer diagnosis (apart from non-melanoma skin cancer), or interview. Women were also considered as menopausal at age 55 years. Women reporting RRSO or hysterectomy as the reason for periods stopping were considered premenopausal until the age at last period. Women reporting periods stopping (due to natural menopause, RRSO or hysterectomy) but with missing age at menopause or age at last period were excluded from the analyses (see STable 2).

RRSO and hysterectomy are initially collected by self-reported questionnaires. When a participant self-reports RRSO (with or without hysterectomy), the study team then confirms these reports with the hospital and/or clinic. The reasons for censoring by menopausal status, are summarised in STable 3. The numbers of women experiencing RRSO at censoring and the numbers of breast cancers diagnosed prior to or at interview by age at diagnosis are shown in Table 1.

**Table 1:** Characteristics of *BRCA1* and *BRCA2* PV carriers and non-carriers in EMBRACE

	*BRCA1 PV carriers*	*BRCA2 PV carriers*	Non-carriers
	*N* = 3,046	*N* = 3,264	*N* = 2,857
Age at interview (years)	46 (13); 45 (19)	48 (13); 48 (20)	46 (14); 45 (20)
Age at interview (years) categories			
< 30	358 (12%)	281 (8.6%)	390 (14%)
30–39	752 (25%)	686 (21%)	672 (24%)
40–49	818 (27%)	835 (26%)	705 (25%)
50–59	647 (21%)	758 (23%)	585 (20%)
60 +	471 (15%)	704 (22%)	505 (18%)
Birth cohort (year)^a^			
< 1940	110 (3.6%)	144 (4.4%)	125 (4.4%)
1940–1949	369 (12%)	431 (13%)	323 (11%)
1950–1959	604 (20%)	739 (23%)	608 (21%)
1960–1969	833 (27%)	861 (26%)	779 (27%)
1970–1979	618 (20%)	604 (19%)	502 (18%)
> = 1980	512 (17%)	485 (15%)	520 (18%)
Age at menarche (years)	12.97 (1.56);13.00 (2.00)	12.90 (1.51); 13.00 (2.00)	12.94 (1.53); 13.00 (2.00)
Unknown age at menarche (N)	35	24	25
Age at menarche (years) categories			
< 12 years	556 (18%)	634 (20%)	552 (19%)
12–14 years	1,996 (66%)	2,151 (66%)	1,859 (66%)
> = 15 years	459 (15%)	455 (14%)	421 (15%)
Unknown	35	24	25
Height (m)	1.641 (0.07);1.63 (0.08)	1.639 (0.07); 1.63 (0.08)	1.636 (0.07);1.63 (0.09)
Unknown height (N)	6	7	6
BMI at interview (kg)	25.6 (5.3); 24.6 (6.4)	25.8 (5.4); 24.6 (6.2)	25.7 (5.4); 24.6 (6.1)
Unknown BMI (N)	26	22	30
BMI at age 18 (kg)	21.49 (3.28); 20.99 (3.38)	21.46 (3.20); 20.91 (3.37)	21.55 (3.41); 20.99 (3.49)
Unknown BMI age 18 (N)	109	101	101
Menopausal status (y/n)			
Premenopausal	2,667 (88%)	2,618 (80%)	2,212 (77%)
Menopausal	379 (12%)	646 (20%)	645 (23%)
Age at menopause (years)	50.04 (4.36); 51 (5)	50.55 (4.31); 51 (5)	50.77 (3.85); 51 (5)
Age at RRSO	46 (40, 52)	48 (43, 56)	45 (40, 50)
RRSO at censoring (N)	310	228	157
Interval between menarche and menopause or censoring (years)			
mean, sd, median, iqr	26.2 (8.5); 26.4 (13.1)	28.3 (8.6); 28.8 (12.8)	28.0 (9.4); 28.9 (15)
Interval between menarche and menopause in menopausal women (years)			
mean, sd, median, iqr	36.9 (4.5); 38 (5.5)	37.6 (4.6); 38 (6)	37.7 (4); 38 (5.5)
range	(13.5—45.0)	(16—45)	(23—45)
(0,30]	35 (9%)	55 (9%)	36 (6%)
(30,35]	80 (21%)	117 (18%)	122 (19%)
(35,40]	179 (47%)	276 (43%)	309 (48%)
(40,45]	83 (22%)	194 (30%)	175 (27%)
Menstrual cycle regularity			
Always regular	1612 (53%)	1807 (56%)	1521 (54%)
Usually regular	1064 (35%)	1125 (35%)	966 (34%)
Never regular	348 (12%)	312 (10%)	348 (12%)
Missing	22	20	22
Menstrual cycle length (categories) among women reporting “always regular” or “usually regular” cycles			
< 26 days	337 (13%)	362 (13%)	325 (14%)
26—27 days	231 (9%)	263 (9%)	225 (9%)
> = 28 days	1988 (78%)	2191 (78%)	1839 (77%)
Missing cycle length (N)	104	105	86
Parity			
Nulliparous	672 (22%)	665 (20%)	696 (24%)
1 live birth	498 (16%)	521 (16%)	450 (16%)
2 live births	1,179 (39%)	1,308 (40%)	1,076 (38%)
3 + live births	697 (23%)	770 (24%)	635 (22%)
Age at first birth (years) categories			
< 20	332 (14%)	342 (13%)	350 (16%)
20–24	717 (30%)	811 (31%)	721 (33%)
25–29	769 (32%)	834 (32%)	663 (31%)
30–34	403 (17%)	446 (17%)	315 (15%)
> = 35	152 (6.4%)	164 (6.3%)	111 (5.1%)
Unknown Pregnancy Age	1	2	1
Breast cancers diagnoses^b^	1346	1401	105
%	44%	43%	3.7%

^1^Mean (SD); Median (IQR); n (%)

^a^For the main analyses birth cohorts after 1960 were combined

^b^Number of breast cancers diagnosed prior to or at interview

Age at menarche was coded as a continuous variable or categorised as age < 12, 12–14 and ≥ 15 years. The interval between menarche and the earliest of menopause and age at censoring (years) was treated as a continuous variable. Women were asked if occurrence of menstrual cycle was always regular, usually regular, or never regular. For women with always regular or usually regular cycles, menstrual cycle length information was categorised as < 26, 26–27 and ≥ 28 days. Parity at baseline was coded as nulliparous, one, two, or three or more live births. Age at first birth was categorised as a continuous or categorical variable (age < 20, 21–25, 26–30, 31–35, ≥ 35 years). Height (m) was treated as a continuous variable. BMI was calculated as weight (kg) divided by height (m) squared.

### Statistical analyses

To explore whether age at natural menopause was influenced by PV carrier status, we carried out linear regression analyses allowing for a censored outcome, using the cens.normal function in the VGAM package in R (https://CRAN.R-project.org/package=VGAM and [35, 36]). Women were censored at the earliest of age at natural menopause, age at RRSO, any cancer diagnosis apart from non-melanoma skin cancer, death, age at interview or age 55 years. This analysis allowed pre-menopausal women (right censored at baseline) as well as post-menopausal women to be included but assumes that carrier status shifts the mean ANM (rather than the proportional hazards assumption made in a Cox regression). We also carried out standard linear regression, including only women experiencing natural menopause, adjusted for birth cohort (as described below), and adjusting for age-group at censoring (in two-year categories from < 40 to ≥ 54 years), the last age at which menopause could be observed.

These analyses were also used to evaluate the association between carrier status and the interval between ANM and AAM, and carrier status.

Linear regression models were used to test for associations between PV carrier status and AAM, menstrual cycle length, height, BMI at interview and BMI at age 18 years. Associations with categorical AAM and menstrual cycle length was also assessed using multinomial logistic regression.

Participants in EMBRACE were recruited from a population undergoing genetic testing. Affected individuals are therefore more likely to be sampled than unaffected individuals. Additionally, there is a higher probability of sampling younger affected individuals. To account for this bias, a weighted cohort method in which affected and unaffected women are assigned different weights in all analyses according to their age at diagnosis, or age at censoring, was used so that the weighted cohort mimicked a true cohort [37, 38]. This method has been shown to provide estimates of relative risk which are close to unbiased [37, 38]. An individual was considered a case if they had had a breast cancer diagnosis prior to or at age at interview, regardless of menopausal status, and otherwise a control. For calculation of weights the person-years for unaffected women were calculated from birth to the first of age at interview or RRM, while the person-years for affected women were from birth to age at breast cancer diagnosis, regardless of menopausal status. Individuals were weighted such that the observed breast cancer incidence rates were consistent with established age-specific incidence rates and relative risk estimates for *BRCA1* and *BRCA2* PV carriers [12, 39, 40] (STables 4 and 5). Non-carriers were not weighted (weight = 1) as the proportion of non-carriers that were affected was small [41].

Analyses of ANM were adjusted by birth cohort (year of birth < 1940, 1940–1949,1950–1959 and ≥ 1960); by parity, with the number of full-term pregnancies categorised as 0, 1, 2, and 3 or more; and by age at the start of first full-term pregnancy, categorised as < 20, 20–24, 25–29, 30–34 and ≥ 35 years. Analyses were carried out clustering for family membership, and robust variance-adjusted confidence intervals reported.

For analyses of AAM, menstrual cycle length, height, BMI at interview and BMI at age 18 years, models were adjusted using a finer categorisation of birth cohort (i.e. splitting the final category into 1960–1969 and ≥ 1970 groups). For AAM, analyses were also adjusted for BMI at age 18 years. When evaluating menstrual cycle length, analyses were also adjusted for age at interview.

All statistical analyses were conducted using R version 4.3.1 and associated packages.

## Results

### Study participants.

A total of 3,046 *BRCA1* PV carriers, 3,264 *BRCA2* PV carriers and 2,857 non-carriers from EMBRACE were included in the analyses. Cohort characteristics and distribution of reproductive risk factors, height and BMI are shown in Table 1. The distribution of age at interview was similar between carriers and non-carriers. Approximately 44% of carriers had been diagnosed with breast cancer at interview, compared with ~ 3.6% of non-carriers.

### Distribution of age at natural menopause among BRCA1 and BRCA2 carriers and non-carriers

Among women included in the analysis, 379 (12%) of *BRCA1* PV carriers, 646 (20%) of *BRCA2* PV carriers and 645 (23%) of non-carriers experienced natural menopause prior to RRSO, a cancer diagnosis (apart from non-melanoma skin cancer) or interview (Table 1).

There was no effect of carrier status on ANM in linear regression analyses allowing for a censored outcome, which included data from both pre- and post-menopausal women (ANM difference = −0.002 (95%CI: −0.401, 0.397), Table 2). The mean ANM was lower among *BRCA1* carriers than non-carriers (50.0 vs 50.8 years respectively) (Table 1), and this difference was statistically significant in linear regression analyses unadjusted for age at censoring (*p* = 0.01, Table 2), including only menopausal women. However, in line with the primary analyses described above, this difference was no longer apparent when analyses were adjusted for the age at censoring, the last age at which menopause could have been observed (ANM carrier vs. non-carrier difference −0.129 years, (95%CI: −0.578, 0.321)) (Table 2).

**Table 2:** Association between age at natural menopause and *BRCA1* and *BRCA2* PV carrier vs non-carrier status

	***BRCA1* PV carriers**				***BRCA2 PV* carriers**			
	estimate	L95 CI	U95 CI	p-value	estimate	L95 CI	U95 CI	p-value
Linear regression among pre and post-menopausal women using cens.normal function in VGAM								
+ Birth cohort	−0.002	−0.401	0.397	0.991	−0.172	−0.531	0.188	0.349
+ Parity + AFB + BMI + AAM^a^	−0.052	−0.451	0.347	0.798	−0.139	−0.500	0.222	0.451
+ Parity + AFB + BMI18 + AAM^a^	0.006	−0.395	0.406	0.977	−0.104	−0.467	0.260	0.576
In women with ANM or end of FUP > = 40 years	0.077	−0.302	0.457	0.69	0.002	−0.334	0.339	0.989
Linear regression among menopausal women only								
+ Birth cohort	−0.700	−1.241	−0.159	0.011	−0.215	−0.686	0.255	0.369
+ Cens agegroup	−0.129	−0.578	0.321	0.574	−0.171	−0.602	0.260	0.437
+ Cens agegroup + Parity + AFB + BMI18 + AAM^a^	−0.113	−0.570	0.343	0.625	−0.074	−0.509	0.361	0.739
+ Cens agegroup + Parity + AFB^b^	−0.098	−0.579	0.383	0.688	−0.058	−0.510	0.393	0.800

*ANM* age at natural menopause, *AAM* age at menarche, *AFB* age at first birth, *BMI* Body Mass Index at baseline, *BMI18* BMI at age 18 years, ‘Cens agegroup’, refers to analyses adjusted for the last age at which menopause could be observed; FUP, follow-up

Analyses were adjusted for birth cohort (categorised as < 1940, 1940–1949,1950–1959, > = 1960) and using weights derived as described in the Methods

^a^carried out on data with no missing information on AAM, BMI or BMI at age 18, parity; AFB and parity were considered as categorical variables

^b^only among parous women with information on AFB; AFB was considered as a continuous variable

Similarly, there was no difference in distribution of age at menopause between *BRCA2* carriers and non-carriers (mean age at menopause among *BRCA2* PV carriers = 50.6 years; linear regression coefficient = −0.172 (95%CI: −0.531, 0.188) (Table 2). Adjustment for BMI, parity and age at first birth did not materially alter the estimates. Results were similar for sensitivity analyses restricting the definition of PV carriers to those carrying only protein truncating variants (PTVs) (STable 6).

### Distribution of age at menarche, menstrual cycle length and reproductive lifespan among BRCA1 and BRCA2 carriers and non-carriers

Mean age at menarche was 12.97 and 12.90 years for *BRCA1* and *BRCA2* carriers respectively, and 12.94 years among non-carriers. There was no statistically significant difference in age at menarche either alone (as a continuous or categorical variable) or after adjusting for BMI (Table 3). There were no statistically significant differences between carriers and non-carriers in menstrual cycle length (in women with always regular or usually regular cycles) (Table 4). The interval between menarche and age at menopause was also similar between carriers and non-carriers in regression analyses allowing for censoring (Table 5).

**Table 3:** Association between age at menarche and *BRCA1* and *BRCA2* PV carrier vs non-carrier status

	***BRCA1* PV carriers**				***BRCA2* PV carriers**			
Linear Regression								
	estimate	L95 CI	U95 CI	p-value	estimate	L95 CI	U95 CI	*p*-value
AAM as continuous variable + Birth cohort	0.068	−0.014	0.150	0.106	−0.071	−0.152	0.010	0.084
AAM as categorical variable—trend + Birth cohort	0.027	−0.004	0.058	0.085	−0.021	−0.052	0.010	0.187
Analyses among women with no missing information on AAM, BMI, and height								
+ Birth cohort	0.064	−0.019	0.147	0.130	−0.076	−0.158	0.005	0.067
+ Birth cohort + BMI18	0.064	−0.018	0.146	0.127	−0.071	−0.152	0.010	0.085
+ Birth cohort + BMI18 + height	0.056	−0.027	0.138	0.186	−0.075	−0.156	0.006	0.069
Multinomial regression (+ Birth cohort)								
	OR	L95 CI	U95 CI	p-value	OR	L95 CI	U95 CI	p-value
< 12 years	1.000				1.000			
12–14 years	1.115	0.974	1.276	0.115	0.940	0.826	1.069	0.344
> = 15 years	1.171	0.980	1.400	0.082	0.884	0.743	1.053	0.167

AAM, Age at menarche; BMI, Body Mass Index at baseline; BMI18, BMI at age 18 years

Analyses were adjusted for birth cohort (categorised as < 1940, 1940–1949,1950–1959, 1960–1969, 1970–1979, > = 1980); and using weights derived as described in the Methods

**Table 4:** Association between menstrual cycle length and *BRCA1* and *BRCA2 PV* carrier vs non-carrier status

	***BRCA1* PV carriers**				***BRCA2* PV carriers**			
Analyses among women with no missing information on AAM, and with always regular or usually regular periods								
Linear Regression (+ Birth cohort)								
	estimate	L95 CI	U95 CI	p-value	estimate	L95 CI	U95 CI	*p*-value
Menstrual cycle length (days)	0.139	−0.017	0.295	0.081	0.030	−0.115	0.175	0.688
Multinomial regression (+ Birth cohort)								
	OR	L95 CI	U95 CI	p-value	OR	L95 CI	U95 CI	p-value
< 26 years	1.000				1.000			
26–27	1.006	0.793	1.278	0.958	1.131	0.897	1.426	0.297
> = 28	1.041	0.883	1.229	0.630	1.045	0.887	1.231	0.597
Analyses among women with no missing information on AAM, BMI, height and parity and always regular or usually regular periods								
Linear Regression (+ Birth cohort)								
	estimate	L95 CI	U95 CI	p-value	estimate	L95 CI	U95 CI	p-value
Menstrual cycle length (days)	0.143	−0.012	0.299	0.071	0.029	−0.117	0.174	0.699
+ AAM + height + BMI + Parity + AFB^a^	0.128	−0.027	0.283	0.106	0.018	−0.127	0.163	0.808
+ AAM + height + BMI18 + Parity + AFB^a^	0.116	−0.040	0.273	0.146	0.007	−0.140	0.154	0.923

AAM, Age at menarche; BMI, Body Mass Index at baseline, BMI18, BMI at age 18 years; AFB, Age at first birth

Analyses adjusted for birth cohort (< 1940, 1940–1949,1950–1959, 1960–1969, 1970–1979, > = 1980); and using weights derived as described in the Methods; analyses were also adjusted for age at interview

^a^ only among parous women with information on AFB; AFB was considered as a categorical variable

**Table 5:** Interval between menopause and menarche and BRCA1 and BRCA2 PV carrier vs non-carrier status

	***BRCA1* PV carriers**				***BRCA2 PV* carriers**			
	estimate	L95 CI	U95 CI	p-value	estimate	L95 CI	U95 CI	p-value
Birth cohort	−0.064	−0.484	0.356	0.765	−0.036	−0.419	0.347	0.853
+ Parity + AFB + BMI^a^	−0.079	−0.498	0.339	0.710	−0.020	−0.403	0.362	0.917
+ Parity + AFB + BMI18^a^	−0.025	−0.445	0.394	0.905	0.001	−0.384	0.387	0.994

AFB, age at first birth; BMI, Body Mass Index at baseline, BMI18, BMI at age 18 years

Linear regression analyses of interval between menopause and menarche in BRCA1 and BRCA2 PV carrier vs non-carriers were carried out using the norm.cens regression (VGAM); and using weights derived as described in the Methods

^a^ only among women with no missing information on AFB, parity or BMI, Parity and AFB treated as categorical variables

### Distribution of height and BMI among BRCA1 and BRCA2 carriers and non-carriers

*BRCA1* PV carriers were slightly taller than non-carriers (mean difference 0.005 m, p = 0.003); for *BRCA2 PV* carriers the difference was 0.002 m *p* = 0.2 (Table 6). In unweighted analyses, the effect was also statistically significant for *BRCA2* (p < 0.05) (STable 7). The effect estimate was similar after adjusting for covariates BMI or BMI at age 18, AAM, height, parity, and age at first birth. There was no difference in BMI at age at interview or at age 18 years between carriers and non-carriers.

**Table 6:** Association between height, and BMI and* BRCA1* and *BRCA2* PV carrier vs non-carrier status

	***BRCA1* PV carriers**				***BRCA2* PV carriers**			
Trait	estimate	L95 CI	U95 CI	p-value	estimate	L95 CI	U95 CI	*p*-value
height (m)								
+ Birth cohort (finer)	0.005	0.002	0.009	0.003	0.002	−0.001	0.006	0.212
+ Birth cohort (finer) + AAM + Parity + AFB^a^	0.005	0.001	0.008	0.011	0.002	−0.001	0.006	0.242
+ Birth cohort (finer) + BMI18 + AAM + Parity + AFB^a^	0.004	0.001	0.008	0.018	0.002	−0.002	0.006	0.294
BMI (kg)								
+ Birth cohort (finer)	−0.116	−0.407	0.175	0.434	0.235	−0.070	0.539	0.131
+ Birth cohort (finer) + AAM + height + Parity + AFB^a^	0.011	−0.274	0.295	0.940	0.272	−0.025	0.569	0.072
BMI at age 18 (kg)								
+ Birth cohort (finer)	−0.009	−0.196	0.179	0.928	0.087	−0.098	0.272	0.357
+ Birth cohort (finer) + AAM + height	0.044	−0.139	0.227	0.637	0.066	−0.114	0.246	0.471

*AAM* Age at menarche, *BMI*, BMI, Body Mass Index at baseline, *BMI18*, BMI at age 18 years; *AFB* Age at first birth

all analyses adjusted for birth cohort (categorised as < 1940, 1940–1949,1950–1959, 1960–1969, 1970–1979, > = 1980); and using weights derived as described in the Methods

^a^Only women with no missing information on age at menarche, BMI, height and parity were included in the analyses; Parity and AFB treated as categorical variable

## Discussion

We compared the distributions of breast cancer risk factors including ANM, AAM, the interval between ANM and AAM, menstrual cycle length, height and BMI in a cohort of *BRCA1* and *BRCA2* PV carriers, and non-carriers, from a large national study.

We found no statistically significant differences in the distributions of any of these traits, apart from height. In unadjusted analyses among women reporting natural menopause, we observed a lower mean ANM in *BRCA1* carriers compared with non-carriers. However, in naïve analyses not accounting for age at censoring, ANM will inevitably be lower in PV carriers, as natural menopause can only be observed if it takes place prior to RRSO. Analyses adjusting for age at censoring (the last age at which menopause could be observed) or allowing for censoring using the ‘norm.cens’ function in R corrected for this phenomenon, and we found no statistically significant difference in ANM when these analytical strategies were applied. The larger correction for *BRCA1* PV carriers is consistent with the higher cancer risk and more frequent and earlier uptake of RRSO.

DNA damage and repair mechanisms are central in the biology of menopause and BRCA1 and BRCA2 proteins play a crucial role in the process of DNA double strand break repair through regulation of homologous recombination. It is therefore biologically plausible that these processes interact to influence ANM in carriers. Case–control analysis in UK Biobank data have reported earlier natural menopause in women harbouring PTVs in *BRCA1* or *BRCA2* [14]. However, the number of carriers in that study were limited (N = 32 *BRCA1* and N = 143 *BRCA2* carriers). In addition, the effects were smaller in Ward et al. [19], after removing women known to have undergone gynaecological surgeries. The same study [14], however, also reported an earlier ANM in carriers of *PALB2* PTVs, an association that was replicated in data from the BRIDGES study (mean ANM difference 1.78 years) [42]. Given the functional similarity between BRCA2 and PALB2, a similar effect on ANM might be expected, so this discrepancy is perplexing.

While only 14% and 21% of carriers experienced natural menopause in EMBRACE, our study included many more PV carriers than Ruth et al. [14] and should be sufficiently powered to detect differences at least of the magnitude estimated using UK Biobank data. For example, the 95%CI for the effect size in the linear regression would exclude a half year earlier (or later) mean ANM in both *BRCA1* and *BRCA2* PV carriers.

Our results highlight that methodological considerations are important in studies to evaluate risk factors in PV carriers, particularly when evaluating the distribution of age at natural menopause. Interventions, including RRSO in PV carriers, complicate interpretation and results may be sensitive to measurement error. Menopause occurs over a period of time and the recording of both the timing and reason for menopause may be inaccurate. The analyses were based only on data gathered at baseline questionnaire, hence the number of women where the information is completely missing is small. Menopausal status at censoring was inferred/‘imputed’ from answers to multiple different questions. However, potential inaccuracies in the reasons given for menopause, and inaccuracies in ages that periods stopped and other events, could lead to misclassification of menopausal status and a regression to the null. Recording of RRSO and cancer diagnoses may also be incomplete or inaccurate and flagging of cancer could be incomplete. A decision to undergo RRSO may be related to family history of ANM or cancer, as has been previously documented. Furthermore, RRSO may have been scheduled close to anticipated menopause.

There are also limitations in the methodology used to assess associations with ANM. As linear regression ignores data on pre-menopausal women, information is lost. It is also possible that recruitment might be influenced by menopausal status, although this seems unlikely since recruitment is largely determined by family history of cancer. Modelling using the cens.norm function was used as the primary analysis as this method overcomes some of these issues, allowing for censoring whilst using all available data. Due to unbalanced sampling due to recruitment through genetics clinics, analyses with differential weighting of cases and controls were carried out. Another limitation is that non-carriers were only followed up until age at interview, and for this reason only information obtained via the baseline questionnaire was used for both carriers and non-carriers.

Future studies providing accurate record linkage to surgeries and medication use, additional confounders including lifestyle factors related to ovarian aging, and more frequent follow-up to identify when women when first experience menopausal symptoms in relation to other life events, will be valuable.

Menarche, on the other hand, takes place well before the development of cancer, RRSO or genetic testing. We found no association between AAM and carrier status, though age at menarche may be inaccurately reported and could be susceptible to recall bias. BMI at baseline is likely to be accurately reported, and we found no difference in the distributions of BMI between carriers and non-carriers. We did, however, find a small but statistically significant difference in height between carriers and non-carriers, *BRCA1* PV carriers being ~ 0.5 cm taller than non-carriers. Measurement of height is likely to be accurate and unbiased. Height is an established risk factor for breast cancer, and many of the biological pathways underlying growth are also relevant to cancer, but to our knowledge this has not so far implicated BRCA-related mechanisms. This observation could be a chance finding. Alternatively, other unmeasured confounding factors (such physical activity or adolescent smoking) might contribute to this association. Of note, the effect for *BRCA2* PV carriers differs between the weighted and unweighted analyses. If replicated it would be interesting to investigate the mechanisms underlying differences in height between carriers and non-carriers, and implications for cancer risk.

A major strength of this study is comparability between carriers and non-carriers, many of whom are family members of carriers. On the other hand, it is possible that non-carriers are not entirely representative of the general population. Known and unknown factors relevant to membership of a PV carrier family, for example higher levels of screening, or healthy volunteer bias could be relevant.

In addition to the intrinsic biological interest, the results of these analyses have practical implications. The BOADICEA model assumes that the baseline distributions of risk factors are independent of genotype. If that were not the case, the model would need to be adapted to allow for genotype-specific distributions. While the results of our study suggest that any association between PV status and ANM is likely to be weak, and we report no association between PV status and AAM or BMI, these results should be evaluated in the context of limitations outlined above inherent in evaluating these questions in PV carriers. Under the assumption of risk-factor/genotype independence, it would be possible to evaluate the interactions between risk factors in population-based studies using case-only analyses, which are more powerful than case–control analyses, particularly for rare exposures such as PV status. Currently, in the BOADICEA model lifestyle/hormonal risk factors are assumed to be associated with the same relative risk in PV carriers as non-carriers. It has proved difficult to obtain sufficient prospective data to evaluate this directly, and such case-only analyses may provide a more powerful basis to evaluate these interactions. This, in turn, should provide a reliable basis for counselling and management of PV carriers.

## Supplementary Information

Supplementary Material 1.

## Data Availability

The datasets generated and/or analysed during the current study are not publicly available, as they potentially include personal data. However, they can be accessed upon reasonable request made to the EMBRACE study Data Access Coordination Committee (embrace@medschl.cam.ac.uk) and the completion of a data sharing agreement.

## Abbreviations

PV: Pathogenic variant
ANM: Age at natural menopause
AAM: Age at menarche
BMI: Body mass index
RRSO: Risk reducing salpingo-oophorectomy
EMBRACE: Epidemiological Study of Familial Breast Cancer
LOF: Loss-of-function
ER: Oestrogen receptor
CI: Confidence intervals
p: P-value
